# Supplementary material for: Defining a TCF1-expressing progenitor allogeneic CD8+ T cell subset in acute graft-versus-host disease
Source: Nat Commun. 2023 Sep 22;14:5869. doi: 10.1038/s41467-023-41357-9 (PMC10516895; doi:10.1038/s41467-023-41357-9)
Supplement: Supplementary file 1 — Supplementary Information [file 41467_2023_41357_MOESM1_ESM.pdf]

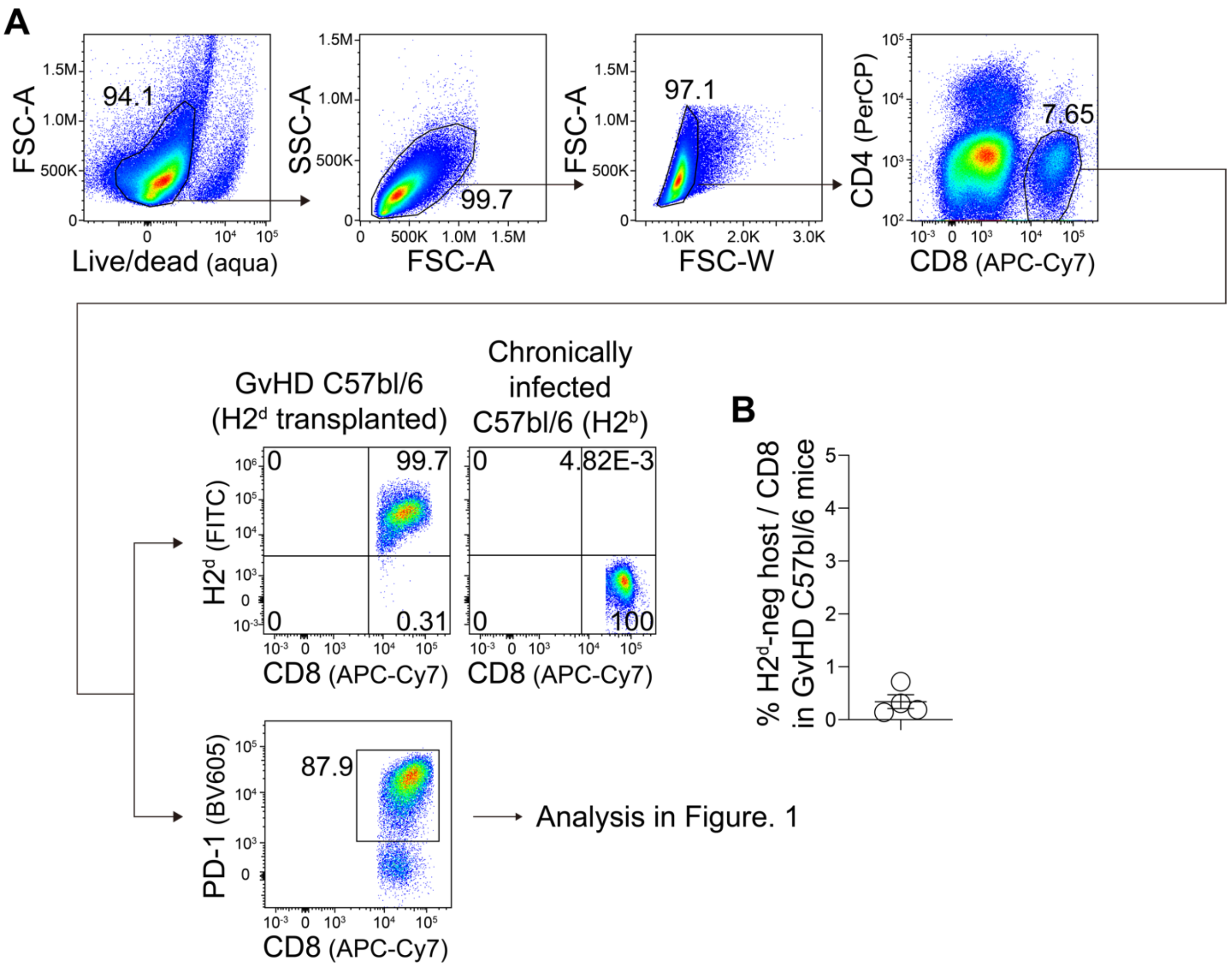

Supplementary Figure 1. Minimal residual population of host CD8$^+$ T cells following allogeneic transplantation

(A) Gating strategy used to characterize CD8$^+$ T cells in mice with acute GvHD. (B) A summary graph showing the frequency of H2^d-negative host CD8$^+$ T cells in the spleen of mice with acute GvHD. Source data are provided as a Source Data file.

A [chronic LCMV, Day 7]

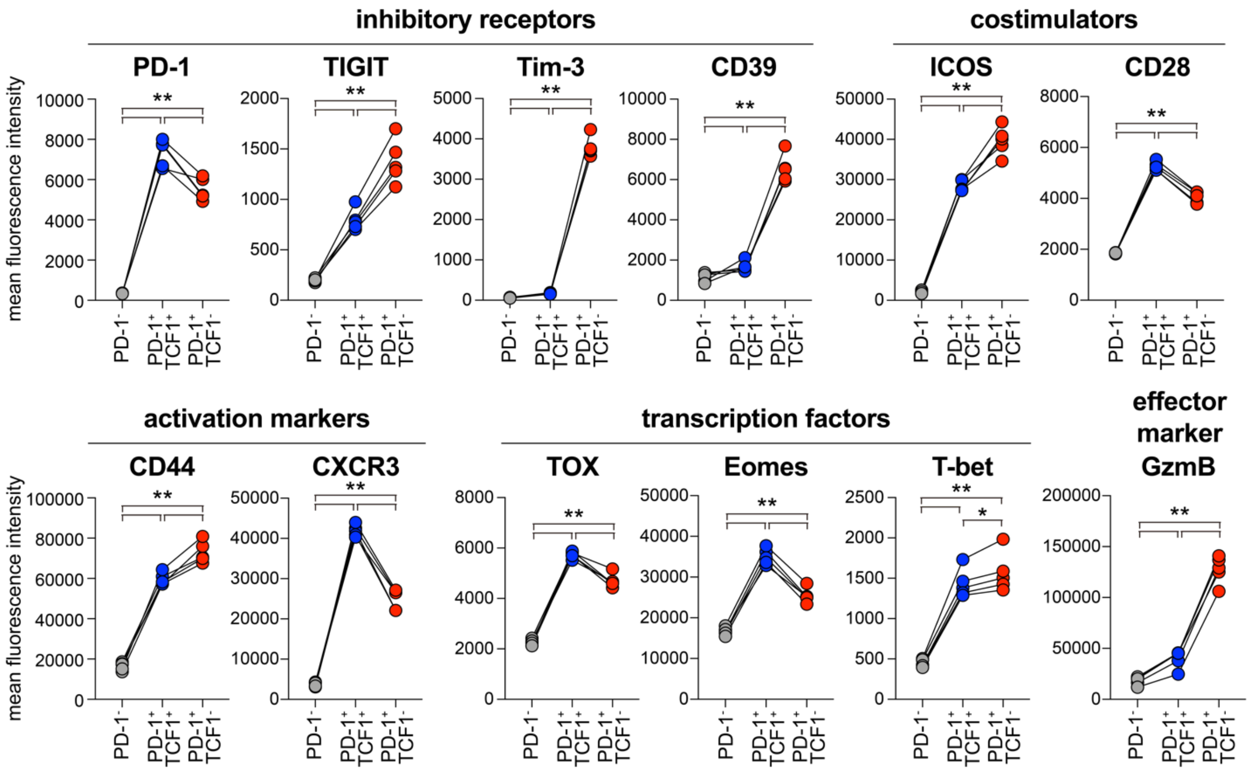

B [acute GvHD, Day 7]

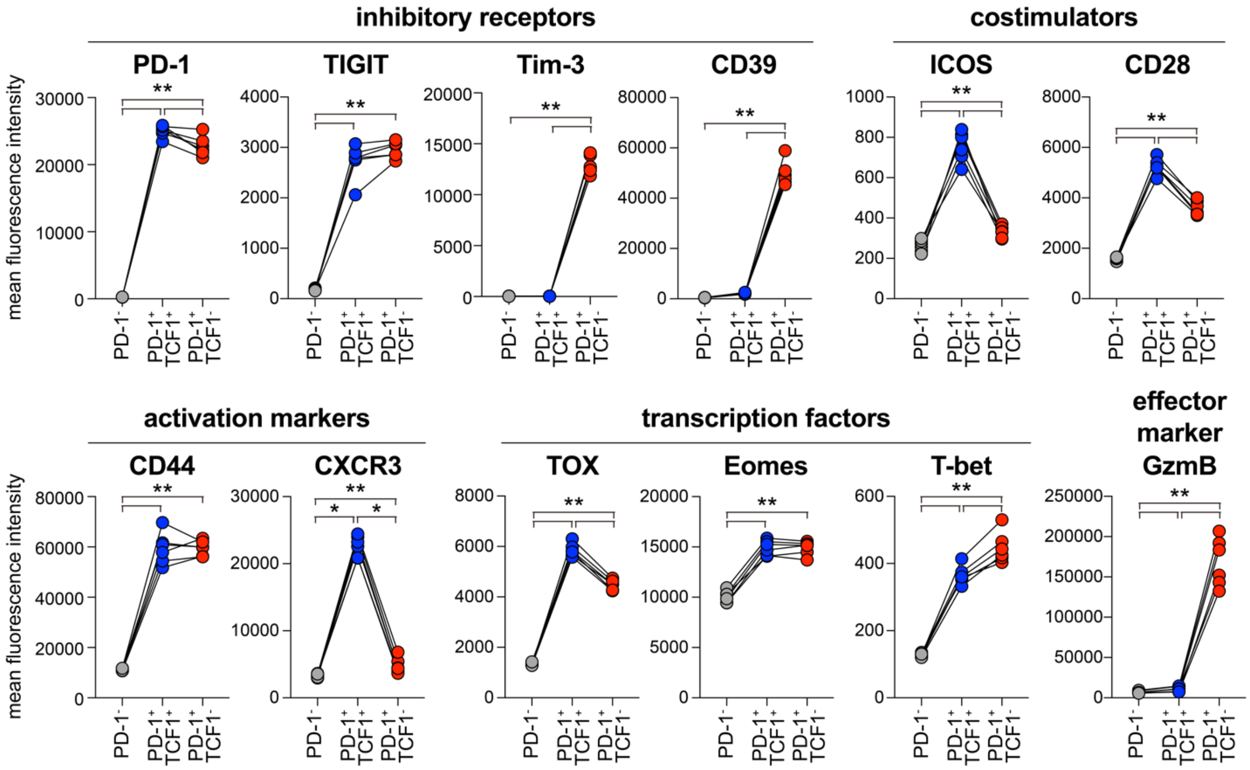

Supplementary Figure 2. Phenotypic similarities of PD-1^{+} CD8^{+} T cell subsets between chronic LCMV infection and acute GvHD

Chronic LCMV infection and acute GvHD were achieved as described in Fig. 1. (A–B) Summary graphs showing the mean fluorescence intensity of proteins, as determined by flow cytometry, on the indicated CD8^{+} T cell subsets in the spleen of chronically infected mice (A) and mice with acute GvHD (B) on day 7 post-infection or post-transplantation. Data are representative of two independent experiments (n=4 or 5 / experiment). Statistical significance was determined by one-way ANOVA with post hoc Tukey's multiple comparisons test (* $p < 0.05$; ** $p < 0.01$). Source data are provided as a Source Data file.

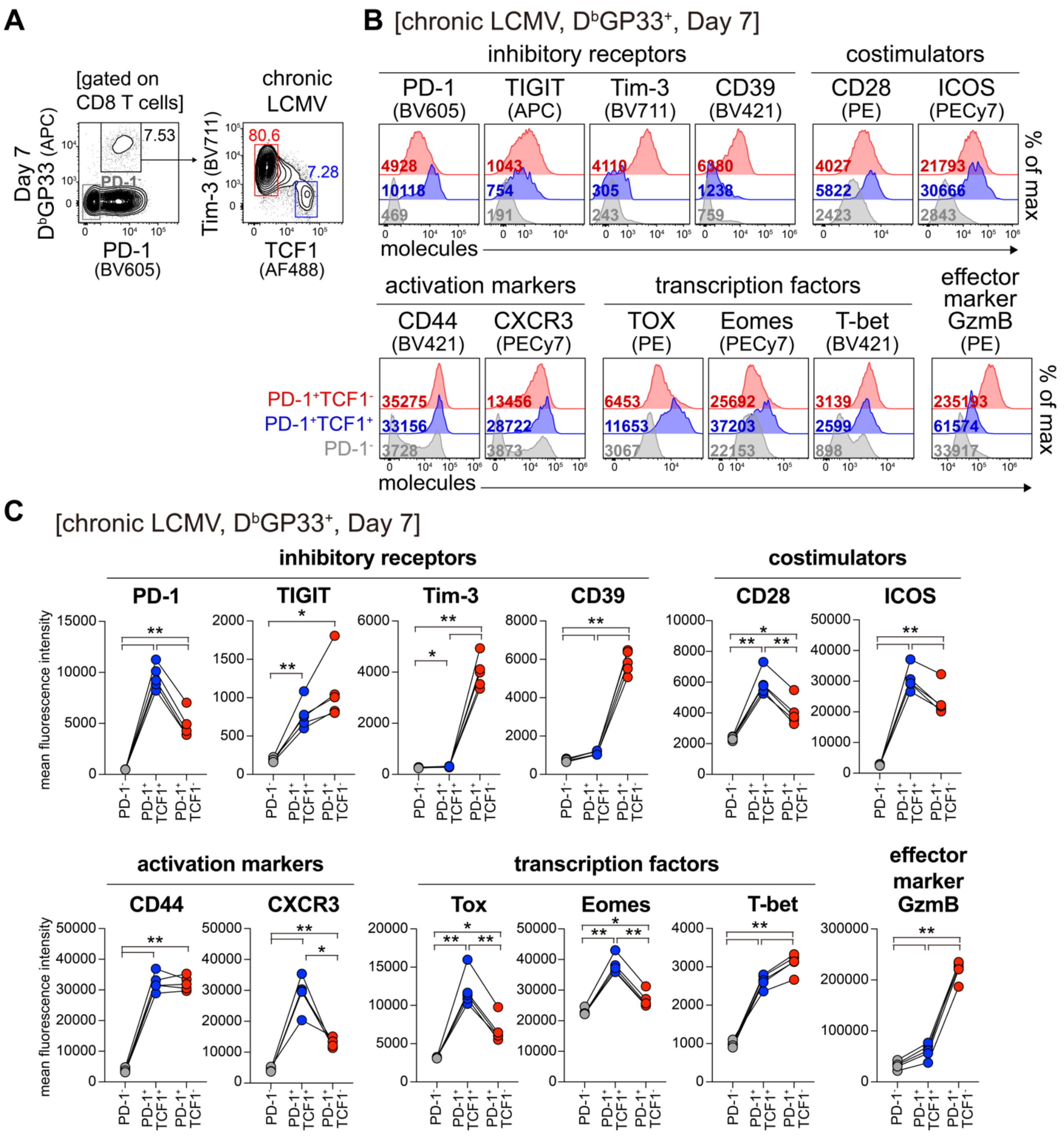

Supplementary Figure 3. Phenotypic similarities of LCMV-specific CD8^{+} T cells to PD-1^{+} CD8^{+} T cells in chronic LCMV infection

Chronic LCMV infection was achieved as described in Fig. 1. (A) Gating strategy used to determine LCMV-specific CD8^{+} T cells using GP33-specific tetramers and their TCF1^{+}Tim-3^{-} and TCF1^{-}Tim-3^{+} subsets in the spleen of chronically LCMV-infected mice at 7 dpi. (B-C) Representative flow plots (B) and summary graphs (C) showing the expression of inhibitory receptors, costimulators, activation markers,

transcription factors, and the effector marker granzyme B on the indicated $CD8^+$ T cell subsets. Data were obtained from a single experiment (n= 5). Statistical significance was determined by one-way ANOVA with post hoc Tukey's multiple comparisons test (* $p< 0.05$; ** $p<0.01$). Source data are provided as a Source Data file.

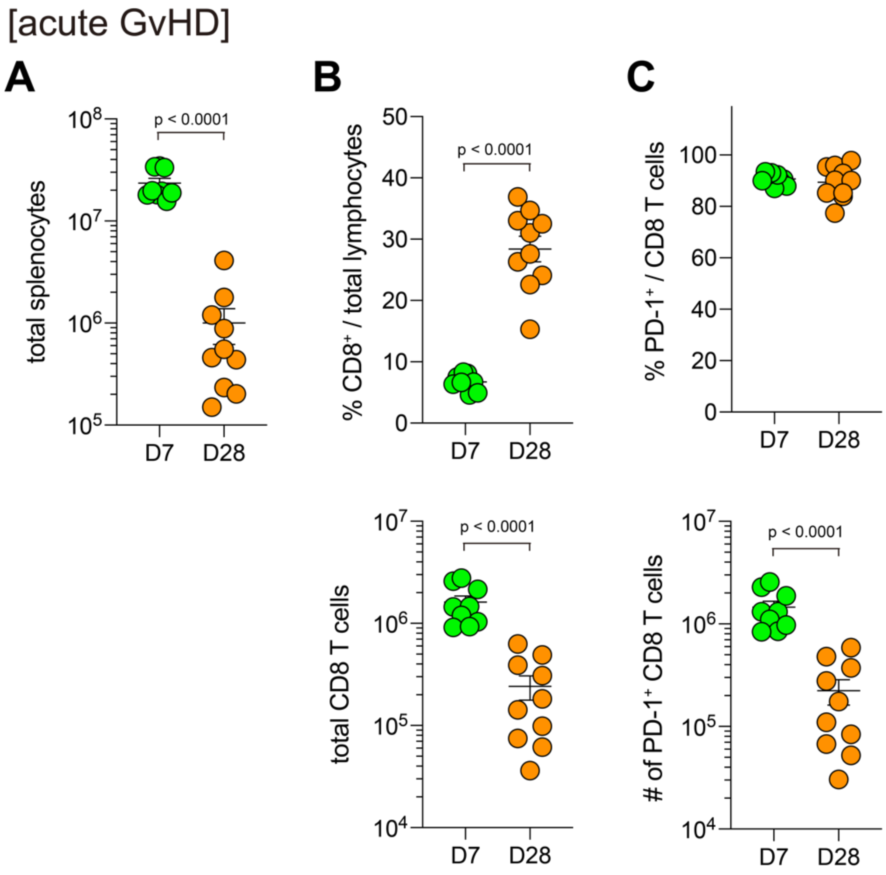

Supplementary Figure 4. Reduced number of total splenocytes, CD8$^+$ T cells, and PD-1$^+$ CD8$^+$ T cells during acute GvHD

Acute GvHD was established as described in Fig. 1. (A) Number of total splenocytes at the indicated time points. (B–C) Frequency (top) and absolute number (bottom) of CD8$^+$ T cells (B) and PD-1$^+$ CD8$^+$ T cells (C) in the spleen at each time point. Data are combined from two independent experiments (n=9 for day 7 and n=10 for day 28), and the mean and SEM were shown. Statistical significance was determined by a two-tailed unpaired t-test. Source data are provided as a Source Data file.

[acute GvHD, Day 28]

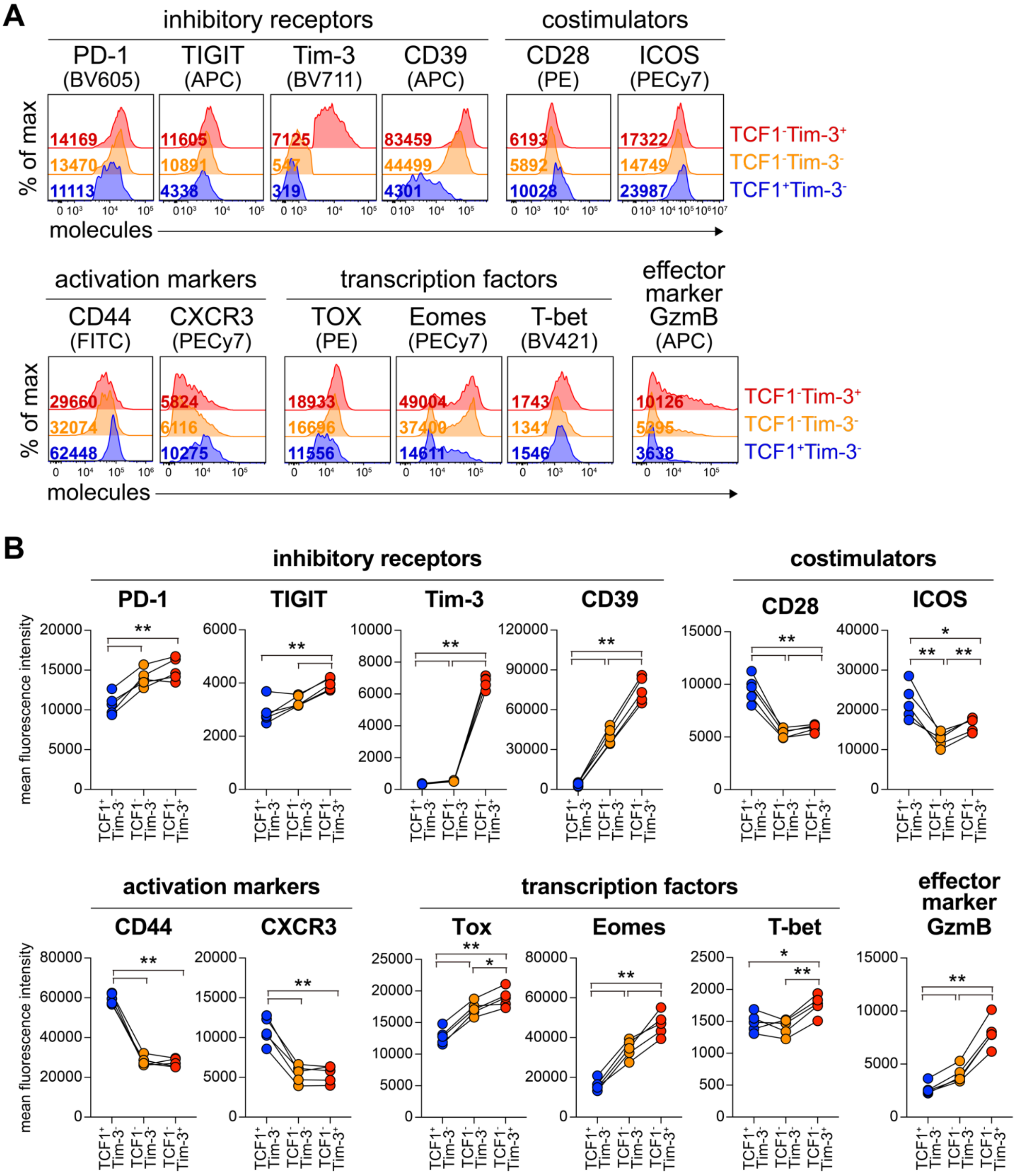

Supplementary Figure 5. Less differentiated phenotypic features of TCF1$^+$PD-1$^+$ alloreactive CD8$^+$ T cells in acute GvHD

Phenotypic analysis of TCF1$^+$Tim-3$^-$, TCF1$^-$Tim-3$^-$, and TCF1$^-$Tim-3$^+$ PD-1$^+$ CD8$^+$ T cell subsets in the spleen of mice with acute GvHD at 28 dpt. (A–B) Representative flow plots (A) and summary graphs (B) showing the expression of inhibitory receptors, costimulators, activation markers, transcription factors, and effector marker granzyme B on the indicated CD8$^+$ T cell subsets. Data are representative of two independent experiments (n=4 or 5 / experiment). Statistical significance was determined by one-way ANOVA with post hoc Tukey's multiple comparisons test (* $p < 0.05$; ** $p < 0.01$). Source data are provided as a Source Data file.

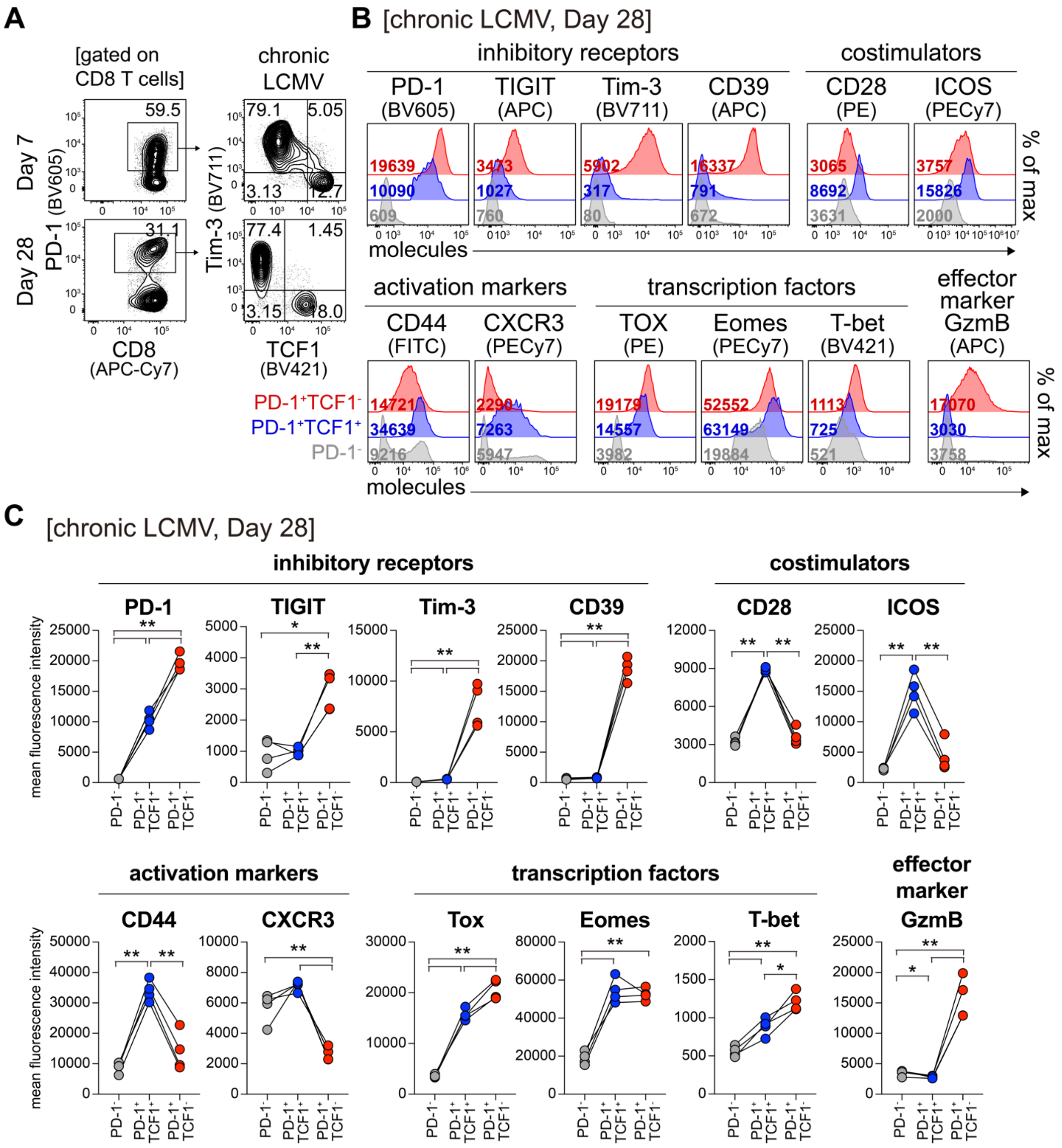

Supplementary Figure 6. Less differentiated phenotypic features of TCF1$^+$PD-1$^+$ CD8$^+$ T cells in chronically infected mice

(A) Gating strategy used to determine TCF1$^+$Tim-3$^-$ and TCF1$^-$Tim-3$^+$ subsets among PD-1$^+$ CD8$^+$ T cells in the spleen of chronically LCMV-infected mice at day 7 and 28 post-infection. (B–C) Phenotypic analysis of TCF1$^+$Tim-3$^-$ and TCF1$^-$Tim-3$^+$ PD-1$^+$ CD8$^+$ T cell subsets as well as PD-1$^-$ CD8$^+$ T cells in the spleen of chronically infected mice at 28 dpt. Representative flow plots (B) and summary graphs (C) showing the

expression of inhibitory receptors, costimulators, activation markers, transcription factors, and the effector marker granzyme B on the indicated $CD8^+$ T cell subsets. Data are representative of two independent experiments (n=4 or 5 / experiment). Statistical significance was determined by one-way ANOVA with post hoc Tukey's multiple comparisons test (* $p< 0.05$; ** $p<0.01$). Source data are provided as a Source Data file.

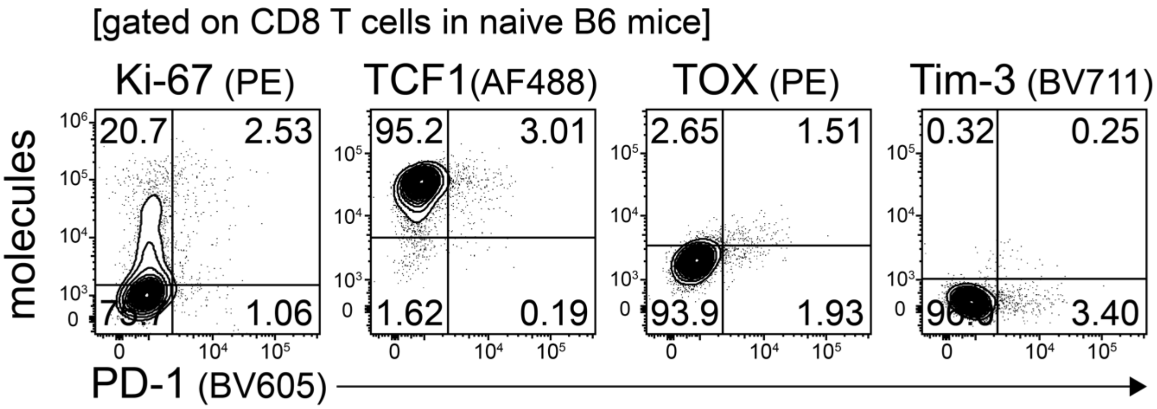

Supplementary Figure 7. Characteristics of splenic CD8$^+$ T cells in naïve mice

Representative flow plots showing the expression of PD-1 vs. Ki-67, TCF1, TOX, and Tim-3 on splenic CD8$^+$ T cells in naïve mice in the same experiment as in Fig. 3A-E.

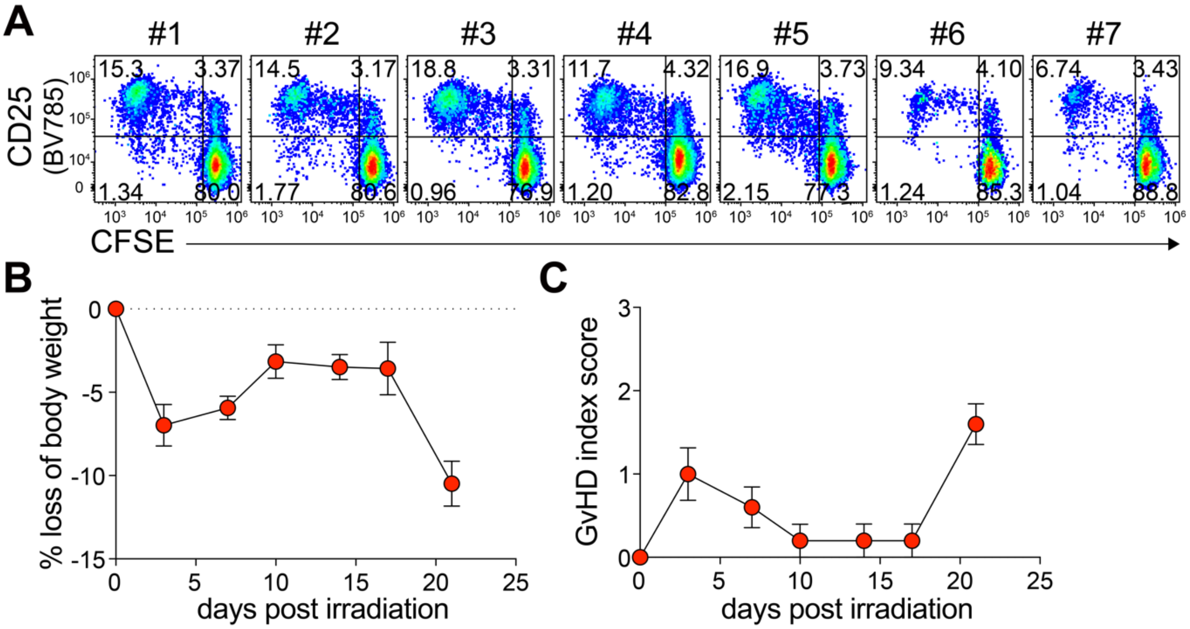

Supplementary Figure 8. Xenogeneic MLR and GvHD induction

(A) Representative flow plots showing the proliferation of hPBMCs responding to the splenocytes of NSG mice by MLR assay. (B-C) Kinetics of body weight loss (B) and GvHD index score (C) during the course of xenogeneic GvHD. Data were obtained from a single experiment (n= 5), and the mean and SEM were shown. Source data are provided as a Source Data file.

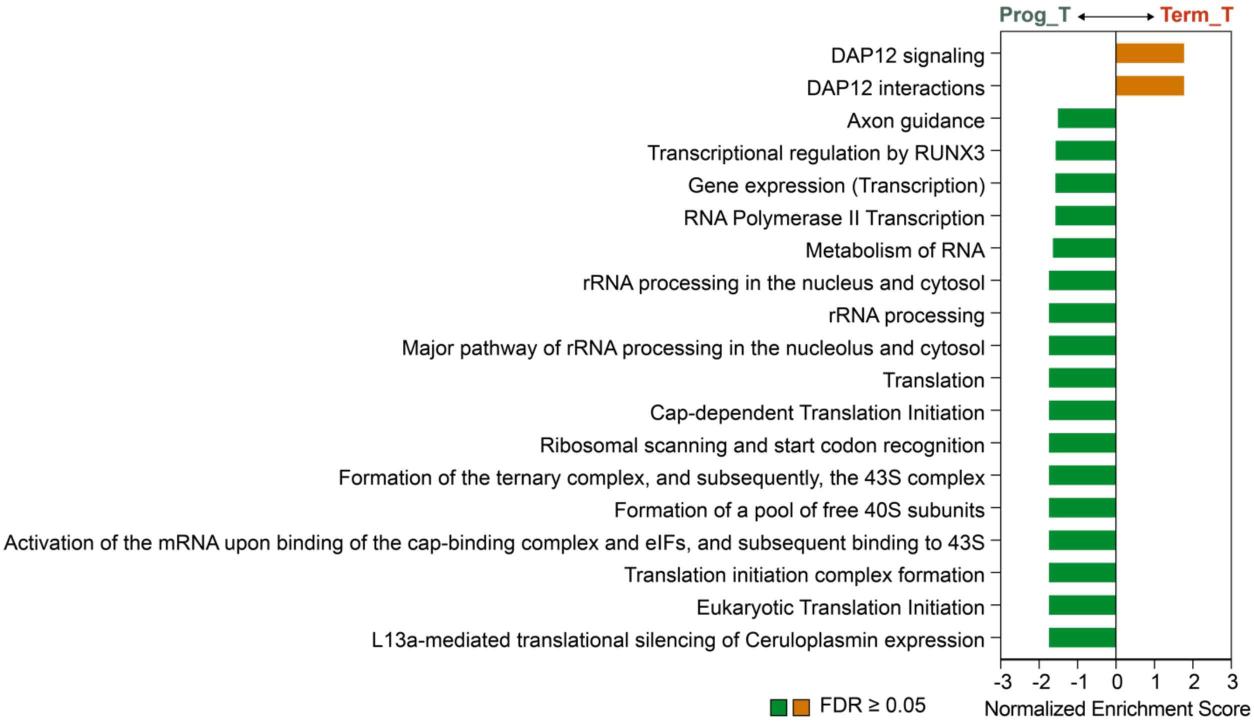

Supplementary Figure 9. Differential transcriptional programs between progenitor and terminally differentiated PD-1$^+$ CD8$^+$ T cell subsets in acute GvHD

Reactome pathway analysis of the highly expressed genes in each subset (false discovery rate, FDR ≥ 0.05).

Source data are provided as a Source Data file.

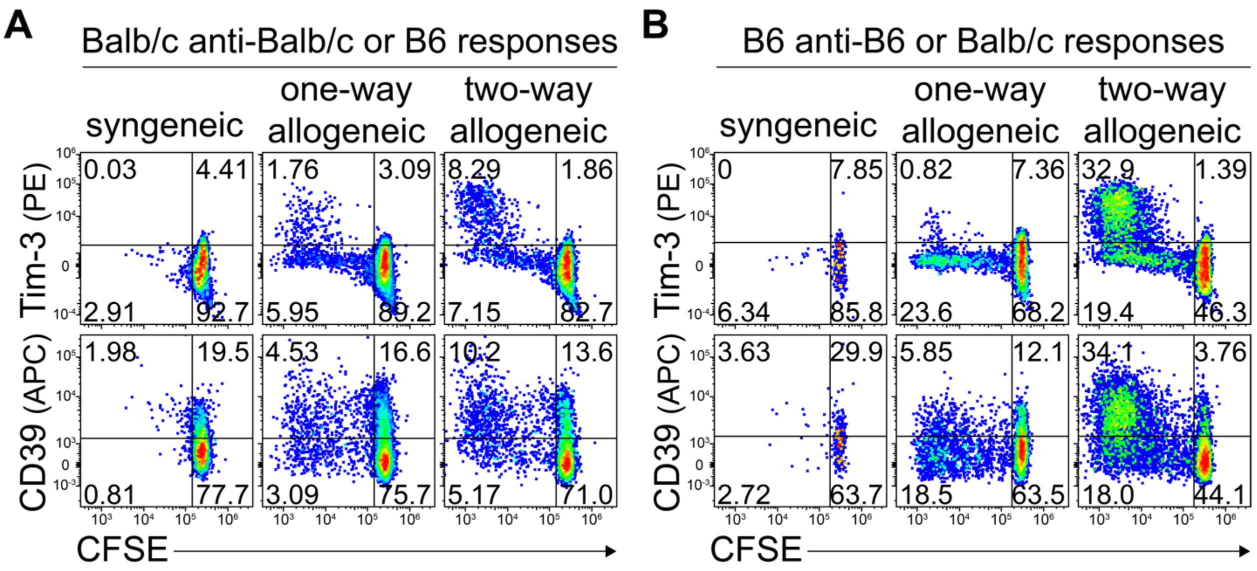

Supplementary Figure 10. Comparison of Tim-3 and CD39 upregulation on allogeneic activated CD8$^+$ T cells between one-way and two-way MLRs

CFSE-labeled naïve CD45.2$^+$ Balb/c or CD45.1$^+$ B6 splenocytes (2x10^5) as responders were incubated with the same number of naïve splenocytes from the same strain (syngeneic), irradiated splenocytes from the other strain (one-way allogeneic), or unirradiated splenocytes from the other strain (two-way allogeneic) as stimulators for 5 days. Responders and stimulators were distinguished in allogeneic MLRs by a congenic marker. **(A–B)** CFSE dilution vs. Tim-3 and CD39 expression on Balb/c (A) and B6 (B) responders. Data are representative of two independent experiments (n=3 / experiment).

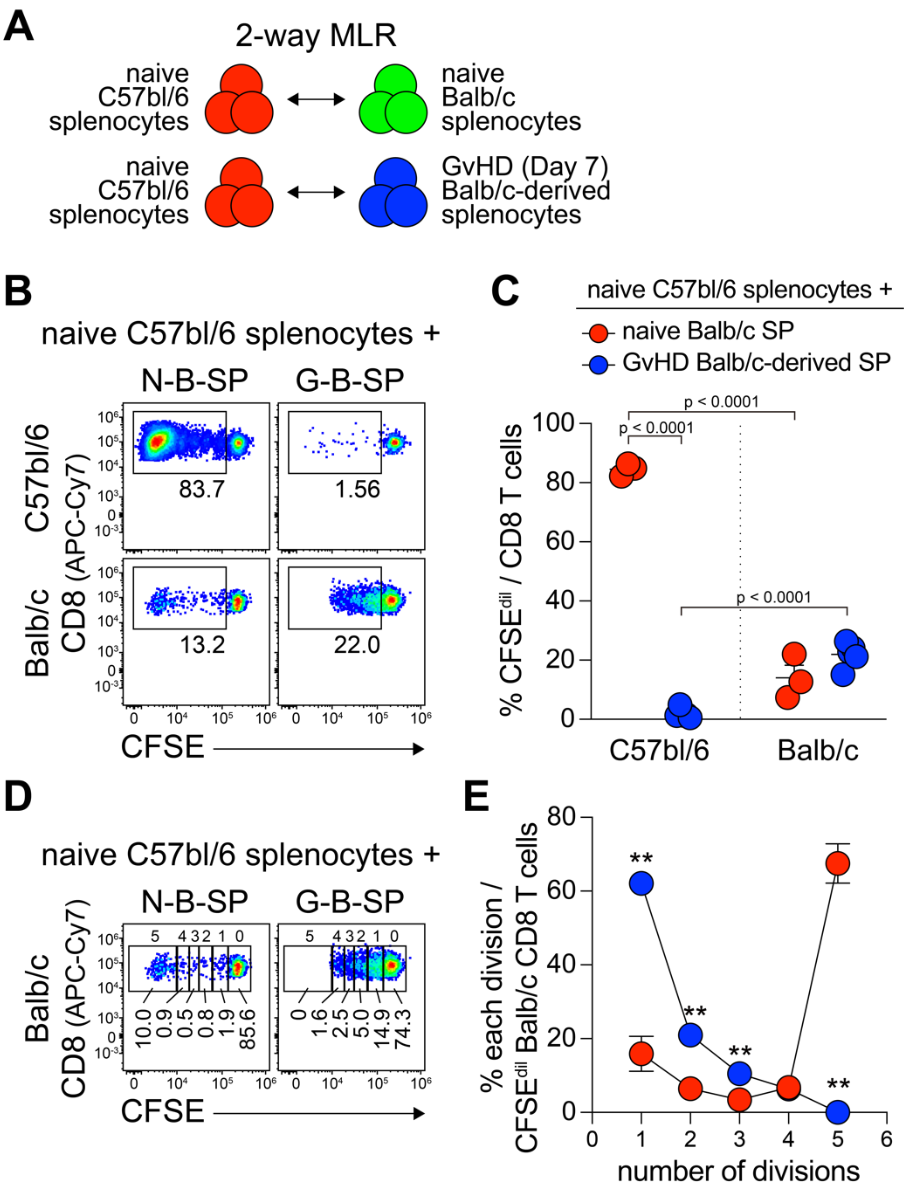

Supplementary Figure 11. Impaired proliferative potential of PD-1^{+} alloreactive CD8^{+} T cells in acute GvHD

(A) Experimental setup: 1) Naïve Balb/c splenocytes (2×10^{5}) and 2) GvHD Balb/c-derived splenocytes (2×10^{5}) were co-cultured with naïve CD45.1^{+} B6 splenocytes (2×10^{5}) for 5 days in two-way MLR. Splenocytes of GvHD B6 recipients of Balb/c T cells were isolated at 7 dpt. (B–C) Representative plots (B) and summary graph (C) showing the proportion of CFSE-diluted cells among CD8^{+} T cells in the indicated conditions. Data were obtained from a single experiment (n=3 of technical replicates), and the mean and

SEM were shown. Statistical significance was determined by one-way ANOVA with post hoc Tukey's multiple comparisons test and was only shown for comparison within each mouse strain or between the same groups. (D) Representative plots of the proportion of $CD8^+$ T cells at each division among Balb/c-derived $CD8^+$ T cells. (E) Summary graph showing the proportion of $CD8^+$ T cells at each division among CFSE-diluted Balb/c-derived $CD8^+$ T cells. Statistical significance was determined by a two-tailed unpaired t-test (** $p<0.01$). Source data are provided as a Source Data file.

Supplementary Table 1. Information about antibodies used for flow-cytometry analysis

Target	Clone	Conjugate	Source	Catalog #	Dilution
Live/dead		Amcyan	Thermo Fisher Scientific	L34966	1:100
Mouse CD28	E18	PE	Biolegend	122010	1:100
Mouse CD39	Duha59	PE-Cy7	Biolegend	143806	1:100
Mouse CD39	Duha59	APC	Biolegend	143810	1:100
Mouse CD4	RM4-5	PerCP	BD Pharmingen	553052	1:75
Mouse CD4	RM4-5	BV421	Biolegend	100544	1:100
Mouse CD44	IM7	BV421	Biolegend	103040	1:200
Mouse CD44	IM7	FITC	BD Pharmingen	553133	1:100
Mouse CD8	53-6.7	APC-Cy7	BD Pharmingen	557654	1:100
Mouse CD8	53-6.7	APC	Biolegend	100712	1:100
Mouse CXCR3	CXCR3-173	PE-Cy7	Biolegend	126516	1:100
Mouse Eomes	Dan11mag	PE-Cy7	Thermo Fisher Scientific	25-4875-82	1:100
Mouse ICOS	C398.4A	PE-Cy7	Biolegend	313520	1:100
Mouse PD-1	RMP1-30	PE	Biolegend	109104	1:100
Mouse PD-1	RMP1-30	PE-Cy7	Biolegend	109110	1:100
Mouse PD-1	29F.1A12	BV605	Biolegend	135220	1:100
Mouse PD-1	29F.1A12	BV421	Biolegend	135218	1:100
Mouse TIGIT	1G9	APC	Biolegend	142106	1:100
Mouse Tim-3	RMT3-23	BV711	Biolegend	119727	1:100
Mouse Tim-3	RMT3-23	PE	Biolegend	119704	1:100
Mouse Tim-3	RMT3-23	APC	Biolegend	119706	1:100
Mouse Tim-3	RMT3-23	BV421	Biolegend	119723	1:100
Mouse/Human TCF1	C63D9	Purified(rabbit IgG)	Cell Signaling Technology	2203	1:100
Mouse/Human TOX	TXRX10	PE	Thermo Fisher Scientific	12-6502-82	1:100
Human CCR7	G043H7	BV421	Biolegend	353208	1:10
Human CD3	OKT3	BV605	Biolegend	317322	1:10
Human CD39	A1	PerCP-Cy5.5	Biolegend	328218	1:10
Human CD45RA	HI100	APC	Biolegend	304112	1:10
Human CD8	HIT8a	APC-H7	BD Pharmingen	566855	1:10
Human PD-1	EH12.2H7	PE-Cy7	Biolegend	329918	1:10
Human TIM-3	F38-2E2	BV711	Biolegend	345024	1:10
Rabbit IgG	Polyclonal	DL488	Abcam	Ab96899	1:1000
Rabbit IgG	Polyclonal	AF647	Abcam	ab150159	1:500
Rabbit IgG	Polyclonal	BV421	BD Biosciences	565014	1:50
