# Supplementary material for: Defining a TCF1-expressing progenitor allogeneic CD8+ T cell subset in acute graft-versus-host disease
Source: Nat Commun. 2023 Sep 22;14:5869. doi: 10.1038/s41467-023-41357-9 (PMC10516895; doi:10.1038/s41467-023-41357-9)
Supplement: Supplementary file 3 — Reporting Summary [file 41467_2023_41357_MOESM3_ESM.pdf]

nature portfolio

Corresponding author(s): Se Jin Im

Last updated by author(s): Aug 6, 2023

Reporting Summary

Nature Portfolio wishes to improve the reproducibility of the work that we publish. This form provides structure for consistency and transparency in reporting. For further information on Nature Portfolio policies, see our Editorial Policies and the Editorial Policy Checklist.

Statistics

For all statistical analyses, confirm that the following items are present in the figure legend, table legend, main text, or Methods section.

n/a	Confirmed	
☐	☒	The exact sample size (*n*) for each experimental group/condition, given as a discrete number and unit of measurement
☐	☒	A statement on whether measurements were taken from distinct samples or whether the same sample was measured repeatedly
☐	☒	The statistical test(s) used AND whether they are one- or two-sided<br>*Only common tests should be described solely by name; describe more complex techniques in the Methods section.*
☒	☐	A description of all covariates tested
☐	☒	A description of any assumptions or corrections, such as tests of normality and adjustment for multiple comparisons
☐	☒	A full description of the statistical parameters including central tendency (e.g. means) or other basic estimates (e.g. regression coefficient) AND variation (e.g. standard deviation) or associated estimates of uncertainty (e.g. confidence intervals)
☐	☒	For null hypothesis testing, the test statistic (e.g. *F*, *t*, *r*) with confidence intervals, effect sizes, degrees of freedom and *P* value noted<br>*Give P values as exact values whenever suitable.*
☒	☐	For Bayesian analysis, information on the choice of priors and Markov chain Monte Carlo settings
☒	☐	For hierarchical and complex designs, identification of the appropriate level for tests and full reporting of outcomes
☒	☐	Estimates of effect sizes (e.g. Cohen's *d*, Pearson's *r*), indicating how they were calculated

Our web collection on statistics for biologists contains articles on many of the points above.

Software and code

Policy information about availability of computer code

Data collection	FACS data: a CytoFLEX flow cytometer (Beckman Coulter)<br>scRNA-seq: Agilent Technologies 4200 TapeStation (Agilent) and HiSeq platform (Illumina)
Data analysis	FACS data: Flowjo v10.8.1<br>Summary graphs and statistical analysis: GraphPad Prism v8.2.1<br>scRNA-seq: Cell Ranger (v 5.0.0), R (v 4.1.2), Seurat (v 4.0.6), ggplot2 (v 3.3.6), ggrepel (v 0.9.1), WebGestaltR (v 0.4.4), fgsea (v 1.21.2), monocle3 (v 1.2.9)

For manuscripts utilizing custom algorithms or software that are central to the research but not yet described in published literature, software must be made available to editors and reviewers. We strongly encourage code deposition in a community repository (e.g. GitHub). See the Nature Portfolio guidelines for submitting code & software for further information.

Data

Policy information about availability of data

All manuscripts must include a data availability statement. This statement should provide the following information, where applicable:

- Accession codes, unique identifiers, or web links for publicly available datasets
- A description of any restrictions on data availability
- For clinical datasets or third party data, please ensure that the statement adheres to our policy

ScRNA-seq data are available at GEO under accession number GSE215315.

Research involving human participants, their data, or biological material

Policy information about studies with human participants or human data. See also policy information about sex, gender (identity/presentation), and sexual orientation and race, ethnicity and racism.

Reporting on sex and gender	Voluntarily donated peripheral blood from healthy individuals was supplied by Geonggi Blood Center, affiliated with the Korean Red Cross. We don't have any information regarding the donors.
Reporting on race, ethnicity, or other socially relevant groupings	We don't have any information regarding the donors.
Population characteristics	We don't have any information regarding donors.
Recruitment	Voluntarily donated peripheral blood from healthy individuals was supplied by Geonggi Blood Center, affiliated with the Korean Red Cross.
Ethics oversight	The protocol was approved by the Institutional Review Board at Sungkyunkwan University School of Medicine (approval number: 2022-08-025-001).

Note that full information on the approval of the study protocol must also be provided in the manuscript.

Field-specific reporting

Please select the one below that is the best fit for your research. If you are not sure, read the appropriate sections before making your selection.

☒ Life sciences ☐ Behavioural & social sciences ☐ Ecological, evolutionary & environmental sciences

For a reference copy of the document with all sections, see nature.com/documents/nr-reporting-summary-flat.pdf

Life sciences study design

All studies must disclose on these points even when the disclosure is negative.

Sample size	Statistical methods were not used to predetermine sample size. Sample sizes were determined based on similar studies in the field, previous experiences, balancing statistical robustness and animal welfare (Im et al Nature (2016), Zhou et al Nat Comm (2020), and others).
Data exclusions	No data were excluded.
Replication	All data were reliably reproduced. The number of repeats and sample sizes are stated in the respective figure legends.
Randomization	Animals were randomly allocated into the groups of chronic LCMV infection and acute GvHD. For ex vivo MLR experiments, cells were sorted from pooled splenocytes, and the well locations for cell culture were randomly arranged. For xenogeneic transplantation, an ex vivo MLR was performed to select individuals highly possessing xenoreactive T cells.
Blinding	Investigators were not blinded to group allocation during experimental setup, data collection, and analysis. No blinding was performed since we did not have the personnel resources to consistently perform blinding.

Reporting for specific materials, systems and methods

We require information from authors about some types of materials, experimental systems and methods used in many studies. Here, indicate whether each material, system or method listed is relevant to your study. If you are not sure if a list item applies to your research, read the appropriate section before selecting a response.

Materials & experimental systems

n/a	Involved in the study
☐	☒ Antibodies
☒	☐ Eukaryotic cell lines
☒	☐ Palaeontology and archaeology
☐	☒ Animals and other organisms
☒	☐ Clinical data
☒	☐ Dual use research of concern
☒	☐ Plants

Methods

n/a	Involved in the study
☒	☐ ChIP-seq
☐	☒ Flow cytometry
☒	☐ MRI-based neuroimaging

Antibodies

Antibodies used

553052, Anti-mouse CD4 PerCP, RM4-5, BD Pharmingen, 1:75
100544, Anti-mouse CD4 BV421, RM4-5, Biolegend, 1:100
557654, Anti-mouse CD8a APC/Cy7, 53-6.7, BD Pharmingen, 1:100
100712, Anti-mouse CD8a APC 53-6.7, Biolegend, 1:100
109104, anti-mouse CD279(PD-1) PE, RMP1-30, Biolegend, 1:100
109110, anti-mouse CD279(PD-1) PE/Cy7, RMP1-30, Biolegend, 1:100
135220, anti-mouse CD279(PD-1) BV605, 29F.1A12, Biolegend, 1:100
135218, anti-mouse CD279(PD-1) BV421, 29F.1A12, Biolegend, 1:100
2203S, TCF1/TCF7 (C63D9) Rabbit mAb, C63D9, Cell Signaling Technology, 1:50 or 1:100
ab96899, Goat Anti-Rabbit IgG DyLight488, Polyclonal, Abcam, 1:1000
ab150159, Goat Anti-Rat IgG AF647, Polyclonal, Abcam, 1:500
565014, Goat Anti-Rabbit IgG BV421, polyclonal, BD Biosciences, 1:50
119727, anti-mouse CD366(Tim-3) BV711, RMT3-23, Biolegend, 1:100
119704, anti-mouse CD366(Tim-3) PE, RMT3-23, Biolegend, 1:100
119706, anti-mouse CD366(Tim-3) APC, RMT3-23, Biolegend, 1:100
119723, anti-mouse CD366(Tim-3) BV421, RMT3-23, Biolegend, 1:100
142106, anti-mouse TIGIT(Vstm3) APC, 1G9, Biolegend, 1:100
143806, anti-mouse CD39 PE/Cy7, Duha59, Biolegend, 1:100
143810, anti-mouse CD39 APC, Duha59, Biolegend, 1:100
122010, anti-mouse CD28 PE, E18, Biolegend, 1:100
313520, anti human/mouse/rat CD278(ICOS) PE/Cy7, C398.4A, Biolegend, 1:100
103040, anti-mouse/human CD44 BV421, IM7, Biolegend, 1:200
553133, anti-mouse CD44 FITC, IM7, BD Pharmingen, 1:100
126516, anti-mouse CD183(CXCR3) PE/Cy7, CXCR3-173, Biolegend, 1:100
12-6502-82, anti-Hu/Mo TOX PE, TXRX10, Thermo Fisher Scientific, 1:100
25-4875-82, anti-Mo EOMES PE/Cy7, Dan11mag, Thermo Fisher Scientific, 1:100
L34966, LIVE/DEAD™ Fixable Aqua Dead Cell Stain Kit, Thermo Fisher Scientific, 1:100

328218, Anti-human CD39 PerCP/Cy5.5, A1, Biolegend, 1:10
329918, Anti-human CD279(PD-1) PE/Cy7, EH12.2H7, Biolegend, 1:10
304112, Anti-human CD45RA APC, HI100, Biolegend, 1:10
566855, Anti-human CD8 APC/H7, HIT8a, BD Pharmingen, 1:10
353208, Anti-human CD197(CCR7) BV421, G043H7, Biolegend, 1:10
317322, Anti-human CD3 BV605, OKT3, Biolegend, 1:10
345024, Anti-human TIM-3 BV711, F38-2E2, Biolegend, 1:10

553991, Rat IgG2b, k isotope control APC, A95-1, BD Biosciences, 1:100
132305, anti-mouse CD132 (common γ chain) PE, TuGm2, BioLegend, 1:100
400607, Rat IgG2b, k isotope control PE, RTK4530, BioLegend , 1:100
C34557, CellTrace™ Violet Cell Proliferation Kit BV421, Thermo Fisher Scientific, final 5M
21888, 5(6)-Carboxyfluorescein diacetate N-succinimidyl ester (CFSE) FITC, Sigma, final 5M

* Antibody used for intravascular
109832, anti-mouse CD45.2 BV421, 104, BioLegend, 3 ug/head

* MACSing kit
130-104-075, CD8a+ T cell isolation Kit, mouse, Miltenyi Biotec

Validation

PerCP Rat Anti-Mouse CD4 : https://www.bdbiosciences.com/ko-kr/products/reagents/flow-cytometry-reagents/research-reagents/single-color-antibodies-ruo/percp-rat-anti-mouse-cd4.553052
Brilliant Violet 421™ anti-mouse CD4 Antibody : https://www.biolegend.com/nl-be/cell-health/brilliant-violet-421-anti-mouse-cd4-antibody-7349?GroupID=BLG4211
APC-Cy™7 Rat Anti-Mouse CD8a : https://www.bdbiosciences.com/ko-kr/products/reagents/flow-cytometry-reagents/research-

reagents/single-color-antibodies-ruo/apc-cy-7-rat-anti-mouse-cd8a.557654
APC anti-mouse CD8a Antibody : https://www.biolegend.com/nl-be/products/apc-anti-mouse-cd8a-antibody-150
PE anti-mouse CD279 (PD-1) Antibody : https://www.biolegend.com/nl-be/products/pe-anti-mouse-cd279-pd-1-antibody-454
PE/Cyanine7 anti-mouse CD279 (PD-1) Antibody : https://www.biolegend.com/nl-be/products/pe-cyanine7-anti-mouse-cd279-pd-1-antibody-3612
Brilliant Violet 605™ anti-mouse CD279 (PD-1) Antibody : https://www.biolegend.com/nl-be/products/brilliant-violet-605-anti-mouse-cd279-pd-1-antibody-7648
Brilliant Violet 421™ anti-mouse CD279 (PD-1) Antibody : https://www.biolegend.com/nl-be/products/brilliant-violet-421-anti-mouse-cd279-pd-1-antibody-7330
TCF1/TCF7 (C63D9) Rabbit mAb : https://www.cellsignal.com/products/primary-antibodies/tcf1-tcf7-c63d9-rabbit-mab/2203
Goat Anti-Rabbit IgG H&L (DyLight® 488) : https://www.abcam.com/products/secondary-antibodies/goat-rabbit-igg-hl-dylight-488-preadsorbed-ab96899.html
Goat Anti-Rat IgG H&L (Alexa Fluor® 647) : https://www.abcam.com/products/secondary-antibodies/goat-rat-igg-hl-alexa-fluor-647-ab150159.html
BD Horizon™ BV421 Goat Anti-Rabbit IgG : https://www.bdbiosciences.com/ko-kr/products/reagents/flow-cytometry-reagents/research-reagents/single-color-antibodies-ruo/bv421-goat-anti-rabbit-igg.565014
Brilliant Violet 711™ anti-mouse CD366 (Tim-3) Antibody : https://www.biolegend.com/nl-be/products/brilliant-violet-711-anti-mouse-cd366-tim-3-antibody-14918
PE anti-mouse CD366 (Tim-3) Antibody : https://www.biolegend.com/nl-be/products/pe-anti-mouse-cd366-tim-3-antibody-2657
APC anti-mouse CD366 (Tim-3) Antibody : https://www.biolegend.com/nl-be/products/apc-anti-mouse-cd366-tim-3-antibody-8238
Brilliant Violet 421™ anti-mouse CD366 (Tim-3) Antibody : https://www.biolegend.com/nl-be/products/brilliant-violet-421-anti-mouse-cd366-tim-3-antibody-13392
APC anti-mouse TIGIT (Vstm3) Antibody : https://www.biolegend.com/nl-be/products/apc-anti-mouse-tigit-vstm3-antibody-7527
anti-mouse CD39 PE/Cy7: https://www.biolegend.com/en-us/products/pe-cyanine7-anti-mouse-cd39-antibody-9645
anti-mouse CD39 APC: https://www.biolegend.com/en-us/products/apc-anti-mouse-cd39-antibody-16388
anti-mouse CD28 PE: https://www.biolegend.com/en-us/products/pe-anti-mouse-cd28-antibody-3778
anti human/mouse/rat CD278(ICOS) PE/Cy7: https://www.biolegend.com/en-us/products/pe-cyanine7-anti-human-mouse-rat-cd278-icos-antibody-6908
anti-mouse/human CD44 BV421: https://www.biolegend.com/en-us/products/brilliant-violet-421-anti-mouse-human-cd44-antibody-7225
anti-mouse CD44 FITC: https://www.bdbiosciences.com/en-us/products/reagents/flow-cytometry-reagents/research-reagents/single-color-antibodies-ruo/fitc-rat-anti-mouse-cd44.553133
anti-mouse CD183(CXCR3) PE/Cy7: https://www.biolegend.com/en-us/products/pe-cyanine7-anti-mouse-cd183-cxcr3-antibody-6169
anti-Hu/Mo TOX PE: https://www.thermofisher.com/antibody/product/TOX-Antibody-clone-TXRX10-Monoclonal/12-6502-82
anti-Mo EOMES PE/Cy7: https://www.thermofisher.com/antibody/product/EOMES-Antibody-clone-Dan11mag-Monoclonal/25-4875-82
LIVE/DEAD™ Fixable Aqua Dead Cell Stain Kit: https://www.thermofisher.com/order/catalog/product/kr/en/L34966
Anti-human CD39 PerCP/Cy5.5: https://www.biolegend.com/en-us/products/percp-cyanine5-5-anti-human-cd39-antibody-9112
Anti-human CD279(PD-1) PE/Cy7: https://www.biolegend.com/en-us/products/pe-cyanine7-anti-human-cd279-pd-1-antibody-6154
Anti-human CD45RA APC: https://www.biolegend.com/en-us/products/apc-anti-human-cd45ra-antibody-684
Anti-human CD8 APC/H7: https://www.bdbiosciences.com/en-us/products/reagents/flow-cytometry-reagents/research-reagents/single-color-antibodies-ruo/apc-h7-mouse-anti-human-cd8.566855
Anti-human CD197(CCR7) BV421: https://www.biolegend.com/en-us/products/brilliant-violet-421-anti-human-cd197-ccr7-antibody-7497
Anti-human CD3 BV605: https://www.biolegend.com/en-us/products/brilliant-violet-605-anti-human-cd3-antibody-7666
Anti-human TIM-3 BV711: https://www.biolegend.com/en-us/products/brilliant-violet-711-anti-human-cd366-tim-3-antibody-10207

anti-human granzyme B antibody (GRB05) was validated by the previous study (Lara M Myers. Et al. A functional subset of CD8+ T cells during chronic exhaustion is defined by SIRPα expression. Nature communication. 2019 Feb 15;10(1):794. doi: 10.1038/s41467-019-08637-9.)

Animals and other research organisms

Policy information about studies involving animals; ARRIVE guidelines recommended for reporting animal research, and Sex and Gender in Research

Laboratory animals	Six- to eight-week-old female C57bl/6 (B6, H2b) and Balb/c (H2d) mice were purchased from Orient Bio (Gyeonggi, Republic of Korea). Eight-week-old female 129/Sv (H2b) mice were purchased from DBL Co. (Chungbuk, Republic of Korea). Eight-week-old male NOD/LtSz-PrkdcscidIL2rytm1Wjl (NSG) mice were purchased from Ja Bio (Gyeonggi, Republic of Korea). CD45.1+ B6 mice were kindly provided by Dr. S. J. Ha (Yonsei University, Seoul, Republic of Korea). We used eight-week-old female CD45.1+ B6 mice and CD45.1+/CD45.2+ B6 mice for experiments.
Wild animals	No wild animals were used.
Reporting on sex	Only female mice were used in murine allogeneic transplants, based on similar studies in the field. Male NSG mice were used for xenogenic transplants based on availability.
Field-collected samples	No samples were collected from the field.

Ethics oversight	All experiments were conducted in accordance with the Institutional Animal Care and Use Committee guidelines of the Sungkyunkwan University School of Medicine.

Note that full information on the approval of the study protocol must also be provided in the manuscript.

Flow Cytometry

Plots

Confirm that:

- [x] The axis labels state the marker and fluorochrome used (e.g. CD4-FITC).
- [x] The axis scales are clearly visible. Include numbers along axes only for bottom left plot of group (a 'group' is an analysis of identical markers).
- [x] All plots are contour plots with outliers or pseudocolor plots.
- [x] A numerical value for number of cells or percentage (with statistics) is provided.

Methodology

Sample preparation	Lymphocyte isolation<br>Lymphocytes were isolated from the blood, spleen and liver as described previously (Wherry et al. (2003) J Virol 77, 4911). Briefly, peripheral blood mononuclear cells were isolated by Histopaque-1077 (Sigma) gradient (800 g at 20 'C for 20min). Spleens were dissociated by passing them through a 70 um cell strainer (Corning). Livers were perfused with pre-cold PBS and homogenized via mechanical disruption. Lymphocytes from livers and lungs were purified by a 44-65% Percoll gradient (800 g at 20 'C for 20min).<br><br>Flow cytometry<br>For cell surface staining, antibides were added to cells at dilutions of 1:50-1:1000 in PBS supplemented with 2% FBS and 0.1% sodium azide for 30 min on ice. Cells were washed 3 times, fixed with Fixation/Permeabilization solution (BD Bioscinces). Intracellular Granzyme B staining (dilution, 1:20) was performed by using BD Cytofix/Cytoperm protocol. For detecting intranuclear proteins, Foxp3 staining buffer set (Thermo Fisher Scientific) was used according to manufacturer's instructions. Dead cells were excluded using Live/Dead<br><br>in vivo intravascular staining<br>For in vivo antibody labeling, 3 ug of BV421-conjugated CD45.1 antibody (BioLegend) was intravenously injected into mice with acute GvHD at days post-transplantation. Peripheral blood mononuclear cells and splenocytes were isolated and used for direct ex vivo staining 3 min after the injection as described previously.<br><br>Cell sorting<br>Cell sorting was performed on a FACS Aria II (BD Bioscience). For single-cell RNA sequencing, PD-1+ CD8 T cells were sorted from mice with acute GvHD at 33–35 days post-transplantation. For ex vivo mixed lymphocyte reaction, PD-1+ CD8 T cells, CD39+ CD8 T cells, and CD39- CD8 T cells were sorted from mice with acute GvHD at 7 days post-transplantation to greater than 96% purity. CD8-depleted Balb/c splenocytes were isolated from naïve mice. Magnetic associated cell sorting (MACS, Miltenyi Biotech, San Diego, CA, USA) was used to isolate total CD8 T cells from mice with acute GvHD and CD8-depleted splenocytes from naïve Balb/c mice.
Instrument	Cell sorting was perfomred by FACS Aria III (BD Biosciences).<br>FACS data were acquired on a CytoFLEX flow cytometer (Beckman Coulter).
Software	FACS data were analyzed using Flowjo v10.8.1.
Cell population abundance	Relative quantification by analyzing data in FlowJo v.10.8.1.<br>The purifies of the sorted cells were more than 95%.
Gating strategy	Lymphocytes were gated on FSC/SSC. Doublets were excluded by using FSC-W/FSC-H. Viable cells were then gated by Live/ Dead fixable dead cell stain kits. After gating CD8 T cells, all other gating strategies for the experiments were provided in the figures.

- [x] Tick this box to confirm that a figure exemplifying the gating strategy is provided in the Supplementary Information.
